# Effect of biomimetic material on stress distribution in mandibular molars restored with inlays: a three-dimensional finite element analysis

**DOI:** 10.7717/peerj.7694

**Published:** 2019-09-12

**Authors:** Junxin Zhu, Danmei Luo, Qiguo Rong, Xiaoyan Wang

**Affiliations:** 1 Department of Cariology and Endodontology, Peking University School and Hospital of Stomatology, Beijing, China; 2 Department of Mechanics and Engineering Science, Peking University, Beijing, China

**Keywords:** Finite element analysis, Biomimetic Materials, Enamel, Inhomogeneous nature

## Abstract

**Background:**

Although biomimetic material has become increasingly popular in dental cosmetology nowadays, it remains unclear how it would affect the restored teeth during chewing. It is necessary to study the influence of biomimetic material on stress distribution in the restored teeth.

**Methods:**

Eight three-dimensional finite element (FE) models were constructed and divided into two groups. Group 1 included the FE model of intact molar, and the FE models of inlay-restored molars fabricated from IPS e.max CAD, Lava Ultimate and biomimetic materials individually. Enamel was considered a homogeneous material. Group 2 included the FE models of intact molar and molars restored with inlays using IPS e.max CAD, Lava Ultimate and biomimetic materials individually, considering enamel as an inhomogeneous material.

**Results:**

In Group 1, compared with that in the intact molar, the maximum tensile stress (MTS) in the occlusal grooves decreased in the inlay-restored molars fabricated from IPS e.max CAD and was concentrated on the cavity floor at the buccal side in the inner dentin around inlay. When Lava Ultimate was selected, MTS decreased in the occlusal grooves and on the cavity floor but increased in the lateral walls. In the restored molar using biomimetic material, the MTS on the cavity floor was distributed more evenly than that in the molar using IPS e.max CAD, and no obvious changes were noted in the lateral walls. The same changes were observed in Group 2. No differences in the stress distribution pattern were noted among the FE models in Groups 1 and 2.

**Conclusions:**

Molars restored with inlays fabricated from biomimetic material exhibit a more uniform stress distribution in the dentin around restoration. The consideration of enamel as a homogeneous tissue is acceptable for analyzing the maximum principal stress distribution in the inlay-restored molar.

## Introduction

For coronal damaged teeth with vital pulp, indirect restorations are becoming increasingly popular with the advantages of superior anatomical morphology and proximal contact compared with teeth restored with composite resin. Metal materials are used much less often than before due to their poor esthetics and ability to adhere to dental hard tissues. In recent years, ceramic materials, such as IPS e.max CAD and Vitablocs Mark II, have become preferred restorative materials given their robust esthetic performance and mechanical properties. However, in vitro experiments found that these materials damage the antagonist enamel (Ludovichetti et al., 2018). In addition, inlay-restored molars fabricated from ceramic materials exhibited inferior fracture resistance due to their high elastic modulus (Soares et al., 2004; Liu, Fok & Li, 2014). As the elastic modulus of CAD-CAM composites (such as Brilliant Crios, Lava Ultimate, and Cerasmart) is similar to that of dentin, they are sometimes selected as restorative materials. Nevertheless, its inferior color stability and wear resistance limit its wide application in restoring the coronal damaged teeth in the complicated oral environment (Vanoorbeek et al., 2010; Acar et al., 2016; Zhi, Bortolotto & Krejci, 2016).

Recently, the bio-anthropomorphic materials have become a popular field of study in biomedicine to restore as much physiological function as possible; furthermore, biomimetic materials with location-dependent elastic modulus values may be produced in the future via new technologies, such as three-dimensional printing. Some studies have assessed the feasibility of the application of functionally graded material in prosthetic dentistry (Zhang et al., 2012; Mahmoudi et al., 2018). Restorative materials with different elastic moduli used in the restored teeth could affect the repair effect, which can be explained by the change in stress distribution partly from the standpoint of mechanics (Magne & Belser, 2003). However, the difference in the stress distribution of teeth restored with indirect restorations fabricated from biomimetic materials and traditional materials remains unclear. Given that caries limited in the occlusal surface are commonly observed in coronal damaged teeth with vital pulp, we analyzed the stress distribution of mandibular first molar restored with inlay made of biomimetic material in Class I cavities via finite element (FE) analysis and contrasted these findings with the stress distributions of those restored with traditional restorative materials.

The mechanical behaviors of different restorative materials used in dental field have been studied for a long time. In recent years, the stress states of restored teeth could be analyzed more precisely than before in the numerical simulation by applying a more realistic load, considering the polymerization shrinkage effect and thermal stress caused by changing temperatures (Ausiello et al., 2017; Srivastava et al., 2018; Tribst et al., 2018). However, enamel was still considered as a homogeneous material in almost all FE studies. Despite the fact, enamel was proven to be an inhomogeneous material in early years. Various studies concluded that the elastic modulus of enamel decreased from the occlusal surface to the enamel-dentin junction (EDJ) (Park et al., 2008a, 2008b; Jeng et al., 2011). Local chemistry and microstructure, especially the former, were identified as the main reasons for this variation in the elastic modulus (Braly et al., 2007). He et al. (2013) further found that the relationship between the elastic modulus of enamel (*E*) and the normalized distance from the location of the enamel to the EDJ(*x*) can be expressed as follows: *E*(*x*) = 111.64*x*^0.18^ (*R*^2^ = 0.94). In addition to the variation from the outer to inner enamel, the elastic modulus of enamel also varies between the lingual and buccal surfaces (Cuy et al., 2002). Although materials with different elastic moduli could influence the stress distribution, to the best of author’s knowledge, no study has specifically focused on the relationship between enamel inhomogeneity and the stress distribution in the restored teeth. Therefore, in addition to the influence of the biomimetic material, we also aimed to study the effect of enamel inhomogeneity on the stress distribution in inlay-restored teeth in the present study, and the following null hypothesis was tested: there is no difference in the maximum principal stress distribution in the inlay-restored mandibular molars using biomimetic material or traditional materials regardless of enamel inhomogeneity.

## Materials and Methods

### Modeling

Both of the ethical permission for the study and the need for verbal consent were approved by the Institutional Review Board of School and Hospital of stomatology, Wuhan University (approval number 2012011).

**Figure 1:**
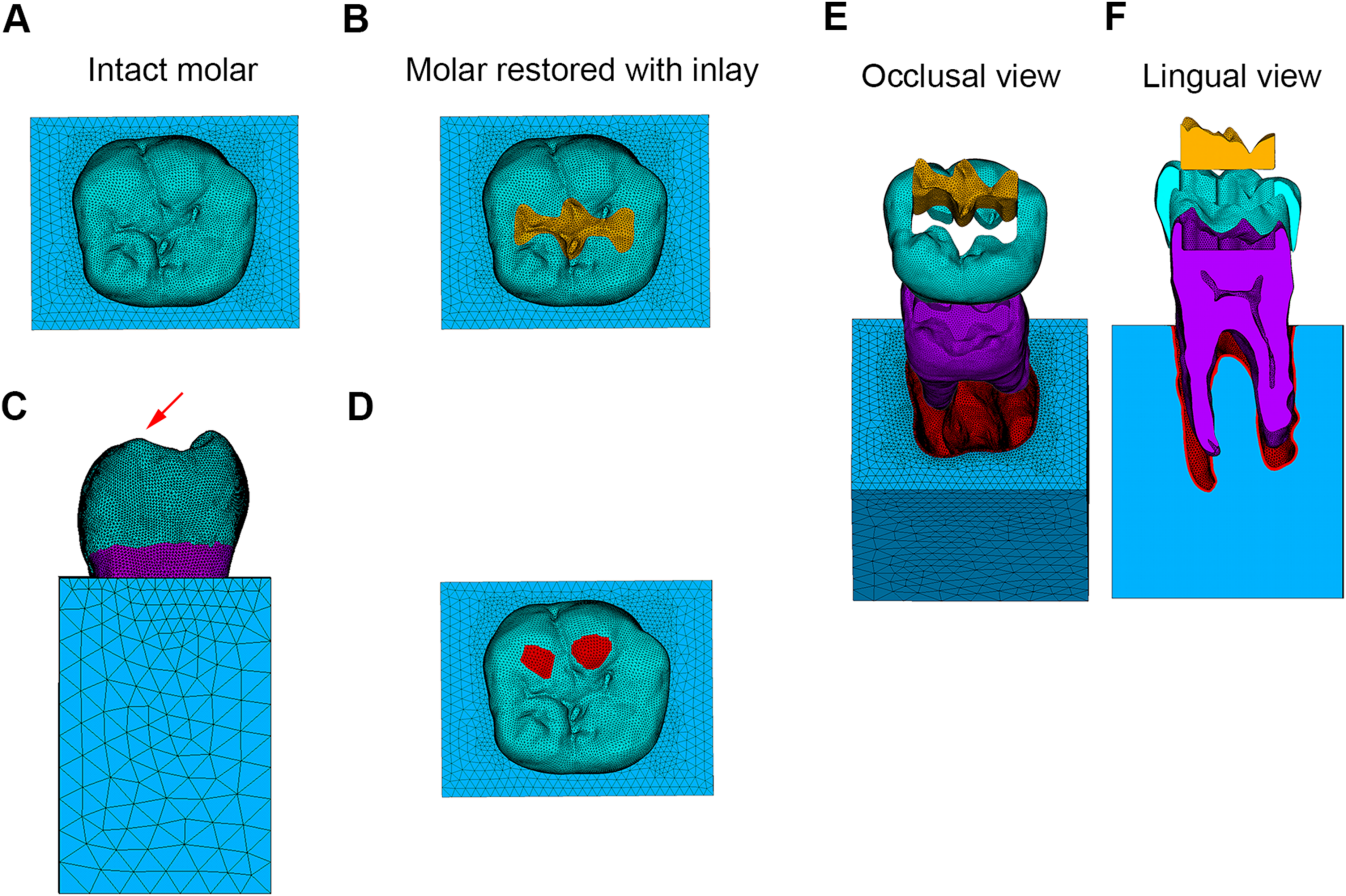
Finite element models and loading conditions. (A) Finite element model of intact molar. (B) Finite element model of molar restored with inlay. (C) Model subjected to oblique load. (D) Loading position. (E) Model of molar restored with inlay in occlusal view. (F) Model of molar restored with inlay in lingual view.

An intact mandibular first molar extracted as a result of periodontal disease was scanned via micro-computed tomography (eXplore Locus SP; GE HealthCare, London, Ontario, Canada). The obtained data (including enamel, dentin, and pulp) were separated using an interactive medical image control system (Mimics 15.0; Materialise, Leuven, Belgium) and were then imported into software (Geomagic Studio; Geomagic Inc, Morrisville, NC, USA) to generate a solid FE model. The portion of dentin below the horizontal plane at the lowest point of the cementoenamel junction was surrounded by a 13 × 17 × 20 mm cuboid, representing the alveolar bone around the root. A 0.2-mm thick periodontal ligament was constructed to connect the molar and alveolar bone. All the dental tissues were considered homogeneous and isotropic with linear elasticity (Table 1). The FE model was designated as the negative control (Fig. 1A). HyperMesh software (Altair Engineering; Troy, MI, USA) was used to mesh the FE model, and a total of 801,606 four-node tetrahedron elements were obtained.

**Table 1:** Material properties.

Material	Elastic modulus (GPa)	Poisson’s ratio	Source
Enamel	84.10	0.33	Dejak, Młotkowski & Langot (2012)
Dentin	18.60	0.31	Dejak, Młotkowski & Langot (2012)
Pulp	0.0068	0.45	Vukicevic et al. (2015)
Periodontal ligament	0.07	0.45	Holmes, Diaz-Arnold & Leary (1996)
Alveolar bone	1.37	0.30	Holmes, Diaz-Arnold & Leary (1996)
IPS e.max CAD	95.00	0.30	Chen et al. (2014)
Lava Ultimate	12.80	0.30	Chen et al. (2014)

To simulate a mandibular molar with caries limited in the occlusal surface restored with inlay, a Class I cavity was built inside the FE model of the intact molar (Fig. 1B). The enamel in Class I cavity was replaced with lithium disilicate ceramic (IPS e.max CAD; Ivoclar Vivadent AG, Schaan, Liechtenstein) and CAD-CAM composites (3M ESPE; Lava Ultimate, St Paul, MN, USA) individually. The dental tissues and restorative materials were considered homogeneous and isotropic with linear elasticity as well (Table 1).

With the assumption that the biomimetic material used in this study could completely recover the assignment of elastic modulus of the original dental tissues, the FE model of an inlay-restored molar fabricated from biomimetic material was created as follows. The inlay was divided into two portions by the structure of EDJ. The portion below the EDJ had the same elastic modulus as the dentin, and the upper portion was set as an inhomogeneous material for which the elastic modulus was consistent with enamel. According to He’s study, the elastic modulus of the graded enamel (*E_i_*) was calculated as follows: *E_i_* = 111.64(*d_i_*)^0.18^, where *d_i_* was the normalized distance from the centroid of the element in the portion of enamel to the EDJ. As the elastic modulus of the upper portion of inlay was set the same to that of enamel, 15,042 four-node tetrahedron elements represented the portion of the inlay limited to enamel were isolated, and the coordinates of each element’s centroid were calculated. In addition, a group of triangular elements was created to fit EDJ, and the coordinates of the nodes belonging to the triangular elements were calculated. According to the coordinates of each tetrahedron element’s centroid and the coordinates of the nodes contained in the group of triangular elements, the node closest to the centroid of the specified element could be identified; furthermore, all the triangular elements, including this node, could be chosen as a set. If the position of the centroid’s projection to the EDJ was located within the triangular element, the distance between the centroid of the tetrahedron element and the triangular element was considered as *d_i_*; otherwise, the distance between the centroid and the closest node was considered as *d_i_*. Other dental tissues (dentin, pulp, periodontal ligament, and alveolar bone) were still considered homogeneous and isotropic with linear elasticity (Table 1).

All the obtained FE models belonged to Group 1. In Group 2, the other conditions of FE models were the same as those in Group 1, with the exception that the enamel was modeled as an inhomogeneous material based on the steps mentioned above.

### Boundary constraint and loading conditions

The degrees of freedom of the nodes contained in the mesial, distal, and basal surfaces of the alveolar bone in *x*, *y*, and *z* directions were set to 0. Given that oblique loads are considered more dangerous to the teeth than the vertical loads (Loney, Moulding & Ritsco, 1995), an oblique load of 250N at 45° to the long axis of the FE models was uniformly applied to the lingual inclined surfaces of the buccal cusps (Figs. 1C and 1D) (Yuan et al., 2016).

### Stress analysis

The ultimate tensile strengths of dental hard tissues are far smaller than their ultimate compressive strengths, so the distributions of maximum principal stress in the FE models were analyzed via Ansys software (Ansys, v16.0; Swanson Analysis Inc, Canonsburg, PA, USA). In the FE model of inlay-restored molar fabricated from IPS e.max CAD in Group 1, the location where the peak maximum tensile stress (MTS) occurred in enamel was determined. Then, the path in the enamel that passed this location in a direction parallel to the long axis of molar was obtained and designated as path 1. Following the direction from the occlusal surface to the EDJ, the maximum principal stress at each node belonging to path 1 was calculated. In the FE model, using the same material belonging to Group 2, the path with a location the same as path 1 was obtained, and the stress at every node belonging to the specified path was also calculated. Paths 2 and 3 were obtained based on the steps mentioned above, representing the vertical path passing the same location in the enamel in the FE models using Lava Ultimate and biomimetic material individually. The maximum principal stress in specified path along the direction from the outer to inner enamel was calculated separately. Furthermore, given that the inner dentin around the cavity in cervical was considered as the stress concentration area according to a previous study (Jiang et al., 2010), the distribution of maximum principal stress in this region was emphatic analyzed in this study.

## Results

**Figure 2:**
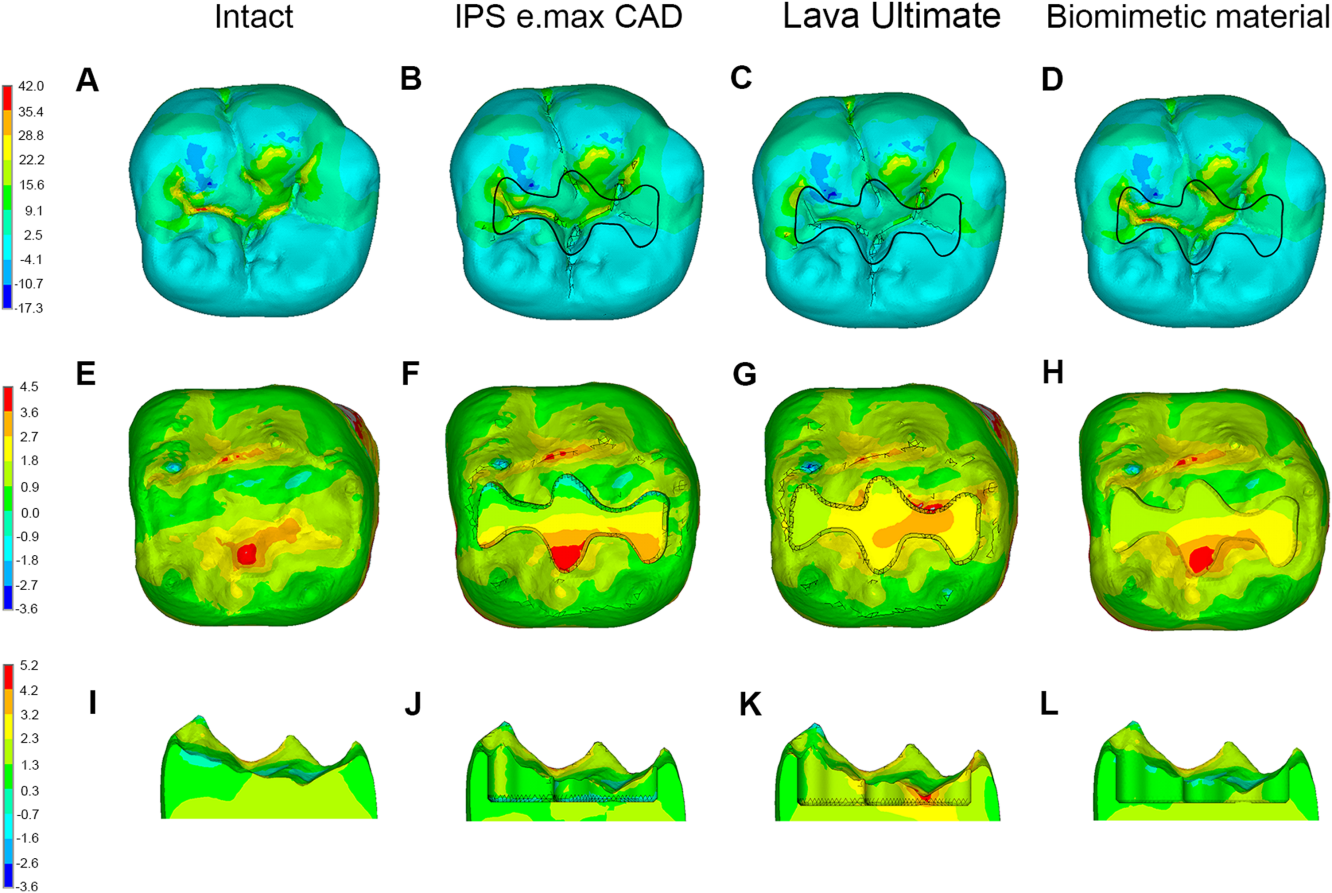
Stress distributions (MPa) in molars in Group 1. (A–D) Maximum principle stress distributions in enamel in intact molar, and in enamel and inlay in molar restored with inlay fabricated from different materials. (E–H) Maximum principle stress distributions in dentin in intact molar, and in molar restored with inlay fabricated from different materials in occlusal view. (I–L) Maximum principle stress distributions in dentin in intact molar, and in molar restored with inlay fabricated from different materials in lingual view.

In the intact molar, the MTS in enamel was concentrated in the occlusal grooves, and the MTS in coronal dentin beneath the enamel was concentrated at the buccal side under the oblique load (Fig. 2). When the mandibular molar was restored using an inlay of the Class I cavity fabricated from IPS e.max CAD, the MTS in the occlusal grooves decreased with no obvious difference in the distribution pattern of maximum principle stress in the remnant enamel. The MTS in the dentin neighboring the inlay was concentrated at the buccal side of the cavity floor and decreased gradually from the buccal to lingual side (Fig. 2).

According to Fig. 2, when the inlay was fabricated from Lava Ultimate, the MTS in the inlay decreased due to its low elastic modulus; however, no obvious difference in the maximum principle stress distribution in the remnant enamel was found. Compared with the inlay-restored molar fabricated from IPS e.max CAD, the stress in the lateral dentin walls of the cavity increased obviously, whereas the MTS on the cavity floor decreased.

When the inlay fabricated from biomimetic material was used to restore the coronal damaged molar, the MTS in the inlay increased with no change in the distribution of maximum principle stress in the remnant enamel. The MTS distribution on the cavity floor was more even than that in the restored molar using IPS e.max CAD with no obvious difference of stress distribution in the lateral dentin walls around the inlay (Fig. 2).

**Figure 3:**
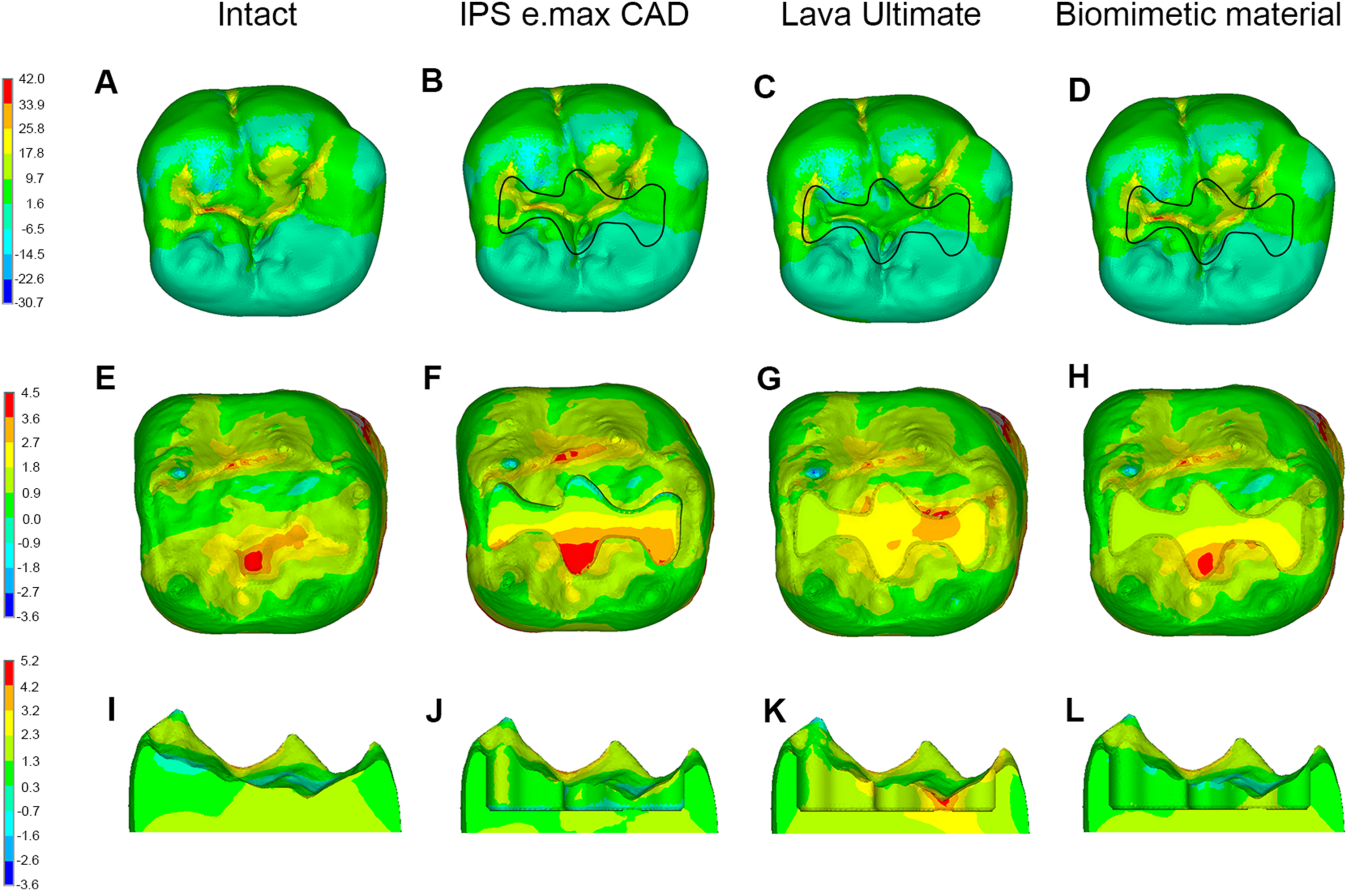
Stress distributions (MPa) in molars in Group 2. (A–D) Maximum principle stress distributions in enamel in intact molar, and in enamel and inlay in molar restored with inlay fabricated from different materials. (E–H) Maximum principle stress distributions in dentin in intact molar, and in molar restored with inlay fabricated from different materials in occlusal view. (I–L) Maximum principle stress distributions in dentin in intact molar, and in molar restored with inlay fabricated from different materials in lingual view.

The same variation tendency of maximum principle stress distributions could also be observed among the FE models in Group 2 (Fig. 3).

**Figure 4:**
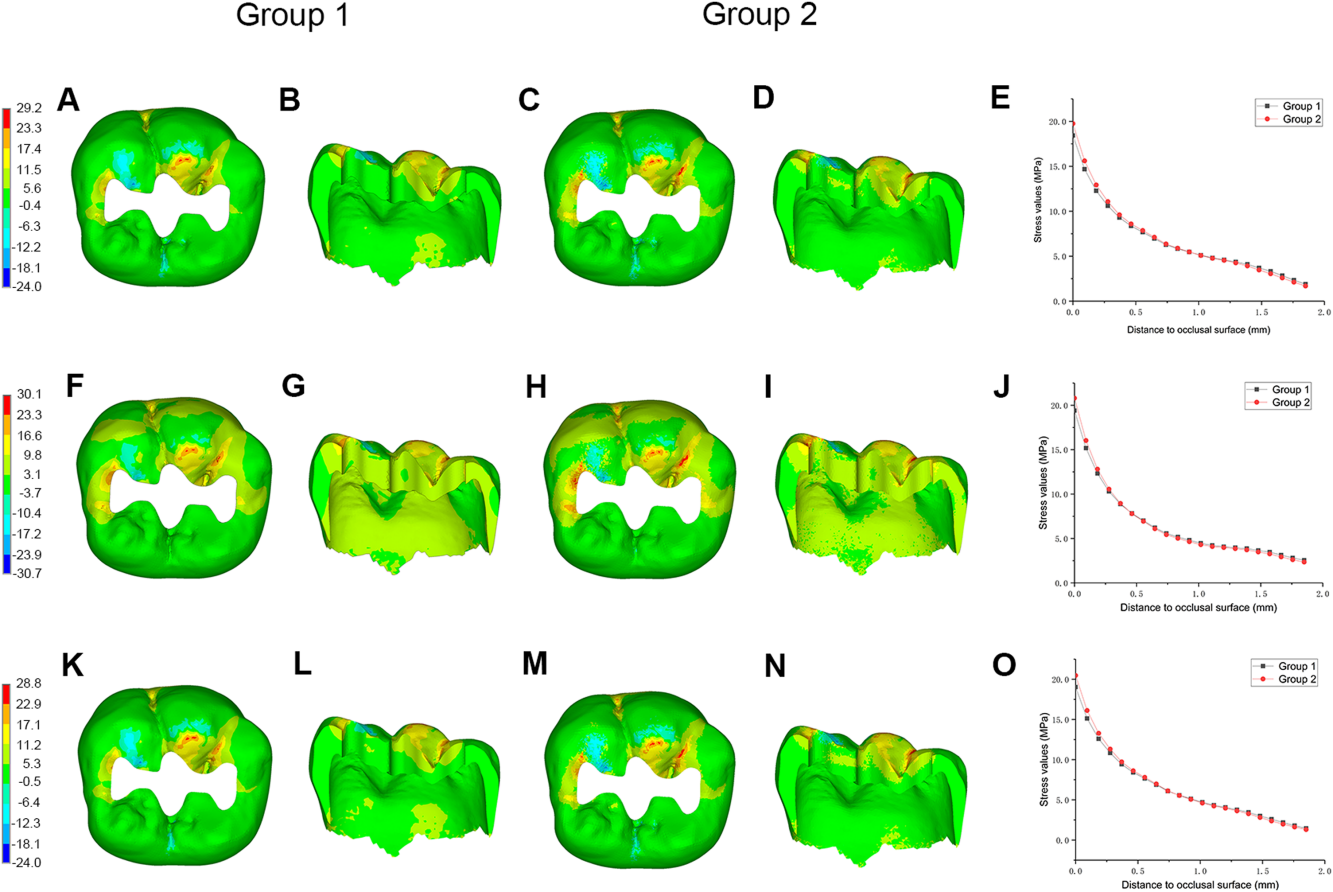
Stress distributions (MPa) in enamel, and stress values in specified path between Group 1 and Group 2. Maximum principle stress distributions in enamel in occlusal and lingual view between Group 1 and Group 2, and stress values in specified path along direction from outer to inner enamel in molar restored with inlay fabricated from (A–E) IPS e.max CAD, (F–J) Lava Ultimate, and (K–O) biomimetic material.

In Group 1, when IPS e.max CAD was chosen to fabricate the inlay, the MTS in enamel was concentrated on the occlusal surface at the mesial and buccal sides and exhibited a falling tendency along the direction from the occlusal surface to the EDJ (Figs. 4A and 4B). Considering the enamel inhomogeneity (Group 2), the MTS in enamel increased at the occlusal surface and decreased adjacent to the EDJ slightly; however, the distribution pattern of MTS was the same as that noted in Group 1. The same change of MTS distributions in enamel was observed between the remaining corresponding models in Groups 1 and 2 (Fig. 4).

**Figure 5:**
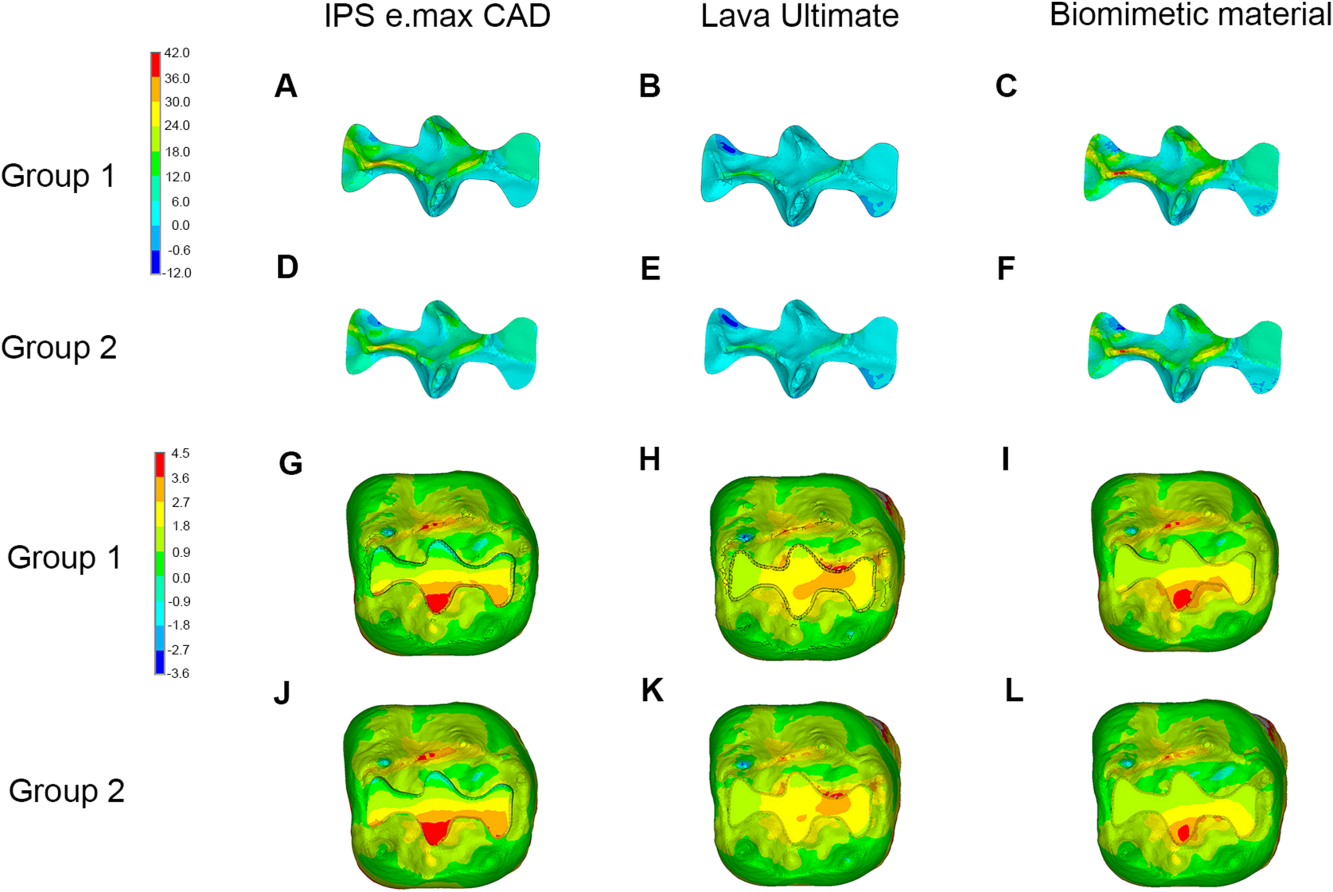
Stress distributions (MPa) in inlay and dentin between Group 1 and Group 2. Maximum principal stress distributions in inlay in molar restored with inlay fabricated from different materials in (A–C) Group 1 and (D–F) Group 2, and maximum principal stress distributions in dentin in molar restored with inlay fabricated from different materials in (G–I) Group 1 and (J–L) Group 2.

Neither the magnitude nor the distribution pattern of maximum principle stress in the inlay or in dentin around the inlay differed between the homogeneous and inhomogeneous models (Fig. 5).

## Discussion

Currently, inlays/onlays are commonly fabricated from ceramic materials due to their perfect esthetic performance and mechanical properties. Although the present study found that the MTS was concentrated in the occlusal grooves in the restored mandibular molar, the inlay made of lithium disilicate ceramic with a tensile strength exceeding 100 MPa could withstand large tensile stress without fracture (Guazzato et al., 2004). In addition, the present study found that in the inner dentin neighboring the inlay, the stress was concentrated on the Class I cavity floor, and the stress was transferred along the axial direction, which was in accordance with a previous study (Jiang et al., 2010). This result indicated that the inner dentin below the inlay suffered from great tensile stress and was thus more prone to fracture in the future.

As one of the emerging restorative materials, CAD-CAM composites with the elastic modulus similar to that of dentin are used in manufacturing of indirect restorations, such as inlays and onlays, in recent years. According to the present study, when the inlay was fabricated from CAD-CAM composites, the MTS in the restoration and on the cavity floor decreased given its low elastic modulus; however, the stress in the lateral dentin walls of the cavity increased obviously due to the large strain of the restoration. Magne & Belser (2003) also found that greater stress was concentrated in the dentin around the restoration when materials with low elastic modulus were selected. As the stress increased, the inlay-restored molars made of CAD-CAM composites exhibit a high risk of repair failure in the future when the residual dentin walls are fragile. In vitro studies also reported that compared with those using ceramic materials, catastrophic fractures were more commonly observed in teeth restored with indirect restorations using laboratory-processed resin (Soares et al., 2004, 2008).

In recent years, bio-anthropomorphic materials have been commonly investigated and developed in the medical field for improved restoration of physiological functions. In this study, we assumed that the vital mandibular molar with coronal loss was restored with an inlay composed of material that could completely recover the original mechanical properties of hard dental tissues. No difference in the stress distribution in enamel was observed regardless of whether traditional or biomimetic materials were used. However, when biomimetic material was selected as the restorative material, the stress was distributed more evenly on the cavity floor compared with the restored molar using ceramic materials, which is considered as a weak region in the inlay-restored molar. The inlay fabricated from layering material dispersed the stress rapidly in the stress-transfer process in the vertical direction, and the dentin beneath the inlay acted as a buffer. Therefore, the stress transferred to the dentin under the restoration decreased. Furthermore, using biomimetic material to fabricate the inlay not only optimized the stress distribution on the cavity floor but also minimized the stress in the lateral dentin walls compared with the restored molar using CAD-CAM composites. This finding can be explained as follows. The biomimetic material used in this study was composed of two parts. The upper portion with high elastic modulus bore greater stress, so the stress transferred to the lower portion was reduced compared with that in the molar restored with CAD-CAM composites. Accordingly, the stress in the lateral dentin walls decreased although large strain occurred in the lower portion of inlay. According to the results, the inlay-restored molars fabricated from biomimetic materials exhibit the best performance in the stress distribution among the restored molars using traditional and biomimetic materials, which are supposed to exhibit superior fracture resistance.

When considering the inhomogeneous nature of enamel, no obvious difference in the distribution pattern of maximum principle stress in enamel was noted regardless of the restorative materials used. However, the stress increased slightly at the occlusal surface and decreased adjacent to the EDJ, indicating that the downward incline of stress along the direction from the outer to inner enamel was steeper when considering the enamel inhomogeneity. This finding can be explained as follows. When enamel was considered to be a homogeneous tissue, the value of the elastic modulus of enamel in every position was the same, and the stress in enamel tended to decrease in the direction from the crown to the root in the stress-transfer process. Once the inhomogeneous nature of enamel was taken into consideration, the outer enamel with a higher elastic modulus had to bear greater stress. Accordingly, the stress transferring to the inner enamel decreased, leading to a more rapid decrease in the transmission of stress in the direction from the outer to inner enamel in total. Interestingly, we also found that considering the enamel inhomogeneity did not change the magnitude or distribution pattern of the maximum principal stress in other dental tissues, such as inlay and dentin. This finding may attribute to the fact that the elastic modulus of elements belonging to the portion of enamel were reassigned during the modeling of inhomogeneous FE models. Regardless of whether the inhomogeneous nature of enamel was considered, the elastic modulus of enamel in homogeneous or inhomogeneous models was still much higher than those of other dental tissues. As a result, reassigning the elastic modulus of enamel would not alter the entire distribution of the maximum principal stress among enamel and other dental tissues. In summary, considering enamel inhomogeneity slightly accelerates the downward incline of stress in enamel along the direction from the outer to inner enamel without change the stress distribution patterns in the dental tissues. Therefore, considering enamel as a homogeneous tissue is acceptable for analyzing the maximum principal stress distribution in the restored molar in order to raise the efficiency of calculation.

With the development of material science, some scholars have begun considering manufacturing complete crowns using functionally graded materials in recent years (Mahmoudi et al., 2018). Traditional modeling approaches cannot be used if the material of the prostheses is inhomogeneous. The present study provides a new method to guide the modeling of inhomogeneous materials, thus making it possible to evaluate the restorative effect of teeth restored with crowns biomechanically.

In the present study, we had not considered the effect of moisture and changing temperature in the complicated oral environment, which might affect the stress distribution. The inhomogeneous nature of enamel was considered only along the direction from the occlusal surface to the EDJ. However, Cuy et al. (2002) found that the elastic moduli of enamel among the cusps, occlusal grooves, and inter-cuspal regions were obviously different. Munari et al. (2015) also concluded that considering the elastic anisotropy of enamel would increase the tensile stress with no change in the pattern of stress distribution. Therefore, further studies are required to consider the inhomogeneous nature of enamel in other directions and its anisotropic nature.

## Conclusions

Molars restored with inlays fabricated from biomimetic material exhibits a more uniform stress distribution in the dentin around restoration. The consideration of enamel as a homogeneous tissue is acceptable for analyzing the maximum principal stress distribution in the inlay-restored molar. However, the inhomogeneous nature of enamel in other directions and its anisotropic nature should be studied in further studies.

## Supplemental Information

10.7717/peerj.7694/supp-1 Supplemental Information 1 Raw data.

## Additional Information and Declarations

### Data Availability

The following information was supplied regarding data availability: The raw data of the FE model is available in the Supplemental File, and can be unzipped into a file in CDB format, which can be opened in Ansys software (Ansys, v14.5; Swanson Analysis Inc, Canonsburg, PA, USA).
